# Super-Critical Accretion onto Stellar Mass Black Holes and Neutron Stars: A Short Review

**DOI:** 10.1007/s11214-026-01318-2

**Published:** 2026-09-09

**Authors:** M. Middleton, G. Lipunova, K. Ohsuga, M. Abramowicz

**Affiliations:** 1 School of Physics & Astronomy, University of Southampton, Southampton, SO17 1BJ UK; 2 Dr. Karl Remeis-Observatory and Erlangen Centre for Astroparticle Physics, Friedrich-Alexander Universität Erlangen-Nürnberg, Sternwartstr. 7 96049, Bamberg, 53121 Germany; 3 Max Planck Institute for Radio Astronomy, Auf dem Hügel 69, Bonn, 53121 Germany; 4 Center for Computational Sciences, University of Tsukuba, Ibaraki, Tsukuba, JP 2018-04-01 Japan; 5 Department of Physics, Göteborg University, Göteborg, 412 96 Sweden; 6 Institute of Physics, Silesian University in Opava, Bezručovo nám, Opava, 13, 746 01 Czech Republic; 7 Nicolaus Copernicus Astronomical Center of the Polish Academy of Sciences, ul. Bartycka 18, Warszawa, 00-716 Poland

**Keywords:** Black holes, Neutron stars, Accretion, Super-Eddington

## Abstract

Super-critical accretion is of both theoretical and observational importance. Theoretically first established by Shakura & Sunyaev in 1973, studies of this accretion regime has since evolved to encompass vital aspects such as advection, surface effects (neutron stars) and the interplay between the plasma, magnetic fields and radiation in full numerical GR-(R)MHD. In this short review of the subject, we present the underlying analytical theory, observational predictions and the role of numerical simulations, with a focus on super-Eddington accretion onto stellar mass black holes and neutron stars. We then discuss observational progress and tensions, and suggestions for how the field might progress in the coming years.

## Introduction

Accretion at or above the Eddington limit (often dubbed super-critical or super-Eddington) represents one of the most dynamic and complex regimes of astrophysical flow. In contrast to the radiatively efficient, geometrically thin discs, super-Eddington accretion flows are characterized by strong radiation pressure, substantial advection of energy with optically-thick accreting material, and powerful mass-loaded outflows. These processes profoundly influence the growth of compact objects, the regulation of feedback into their environments, and the interpretation of a wide range of luminous cosmic sources — from ultraluminous X-ray sources (ULXs) and tidal disruption events (TDEs) to the early growth of supermassive black holes (SMBHs) in quasars. Understanding the physics of super-critical accretion has therefore become a cornerstone of modern high-energy astrophysics.

The theoretical foundations of this field were laid in the 1960s and 1970s, when the concept of an Eddington luminosity — a balance between radiation pressure and gravitational attraction — was applied to accreting compact objects. The self-consistent description of accretion discs was suggested with a self-regulating mechanism, where radiation pressure drives strong winds (Shakura and Sunyaev 1973). In parallel, the recognition that photons can be trapped and advected inward in optically thick flows (Begelman 1979; Abramowicz et al. 1988) led to the development of the so-called slim disc model. These theoretical frameworks introduced the key elements that continue to underpin modern discussions: photon trapping, advection, anisotropic radiation fields, and mass loss through winds.

Subsequent decades have seen rapid advances on both numerical and observational fronts. With the advent of multi-dimensional radiation (magneto-)hydrodynamic (RHD/RMHD) simulations, it became possible to model the intricate coupling between radiation, plasma, and magnetic fields in regimes where radiation pressure dominates. These studies provide new insights on the generation of collimated outflows and relativistic jets and predictions for observational characteristics. The inclusion of general relativity, magnetic fields, and realistic radiation transfer schemes has changed our understanding of the structure and efficiency of these flows, allowing us to include components in theoretical models that are not accessible to analytics: non-trivial geometry, black hole spin, and magnetic state (SANE versus MAD).

On the observational side, the identification of ULXs in nearby galaxies provided compelling evidence for persistent sources with apparent luminosities exceeding the Eddington limit for stellar-mass black holes. The subsequent discovery of pulsations in several ULXs demonstrated that even neutron stars — with solid surfaces and strong magnetic fields — can host super-Eddington accretion, demanding models that incorporate magnetospheric truncation of discs and columnar accretion of plasma. Observations with modern X-ray missions have revealed powerful winds and ultrafast outflows in many ULXs and TDEs, providing direct signatures of the feedback mechanisms long predicted by theory. Together, these results have motivated a unification framework in which the apparent diversity of luminous accreting sources arises primarily from viewing-angle effects and mass-scaling, rather than from fundamentally distinct physical regimes.

Super-Eddington accretion is not merely a laboratory for studying radiation hydrodynamics — it is a process of cosmological significance. The existence of billion-solar-mass black holes at redshifts z>6 implies that black holes must have experienced phases of rapid, possibly supercritical, growth in the early Universe. Similarly, transient super-Eddington episodes are now recognized in TDEs, where the disrupted stellar debris briefly forms an optically thick, radiation-driven disc. In Galactic contexts, ULXs and their associated nebulae provide nearby analogues where the interplay between accretion, outflow, and radiative feedback can be directly observed. Thus, insights gained from super-Eddington flows around stellar-mass objects inform our understanding of SMBH evolution and feedback in galaxies.

Despite remarkable progress, fundamental questions remain open. The relative importance of advection versus wind-driven mass loss, the efficiency of angular momentum transport, and the role of magnetic fields in regulating the accretion rate and jet production are still debated. Numerical simulations, though increasingly sophisticated, face challenges in bridging the vast range of spatial and temporal scales between the innermost flow and its large-scale environment, as well as in accurately treating radiative transfer and multi-temperature plasma physics. Observationally, the geometry and inclination-dependence of emission remain key uncertainties, as does the physical origin of quasi-periodic oscillations (QPOs) and other timing features observed in ULXs and TDEs. Addressing these challenges will require a close synergy between theory, simulation, and observation, leveraging next-generation instruments such as XRISM, NewAthena, and SKA.

This review aims to provide a concise yet comprehensive overview of the physics and observational manifestations of super-Eddington accretion. We begin with the analytical foundations and historical development of theoretical models, followed by a discussion of their numerical realization in multidimensional simulations that incorporate radiation and magnetic fields. We then summarize the observational evidence for super-Eddington accretion across different astrophysical contexts, highlighting both the successes and limitations of current interpretations. Finally, we outline key outstanding problems and directions for future research, emphasizing the need for an integrated approach that combines simulation, spectral modeling, and time-domain astronomy. By synthesizing results from across the mass scale ---- from neutron star ULXs to accreting supermassive black holes – we aim to delineate the common physical principles governing accretion beyond the Eddington limit.

## The Physics of Supercritical Discs

### Analytical Approaches

The discovery of the extragalactic nature of quasars in the 1960s led to the suggestion that their enormous energy output is the result of radiation emitted by matter accreting onto a supermassive black hole, and Lynden-Bell (1969) attributed this process to disc accretion. Salpeter (1964) and Zeldovich and Novikov (1964) first noted that the radiation pressure from the galaxy’s nucleus can expel surrounding matter outward, thereby regulating the core’s mass and luminosity. In the spherical case, the balance of radiation pressure and gravitational pull defines the Eddington luminosity:

1 $$ \frac{L_{\mathrm{Edd}} \sigma _{\mathrm{T}}}{4\pi \,r^{2}\,c} = \frac {m_{\mathrm{p}}\, G\,M }{r^{2}}, \quad L_{\mathrm{Edd}} \approx 1.25 \times 10^{38} (M/M_{\odot})~ \mathrm{erg/s}\, , $$

where $\sigma _{\mathrm{T}}$ is the cross section for Thomson scattering by electrons and $m_{\mathrm{p}}$ is the proton mass.

**Fig. 1:**
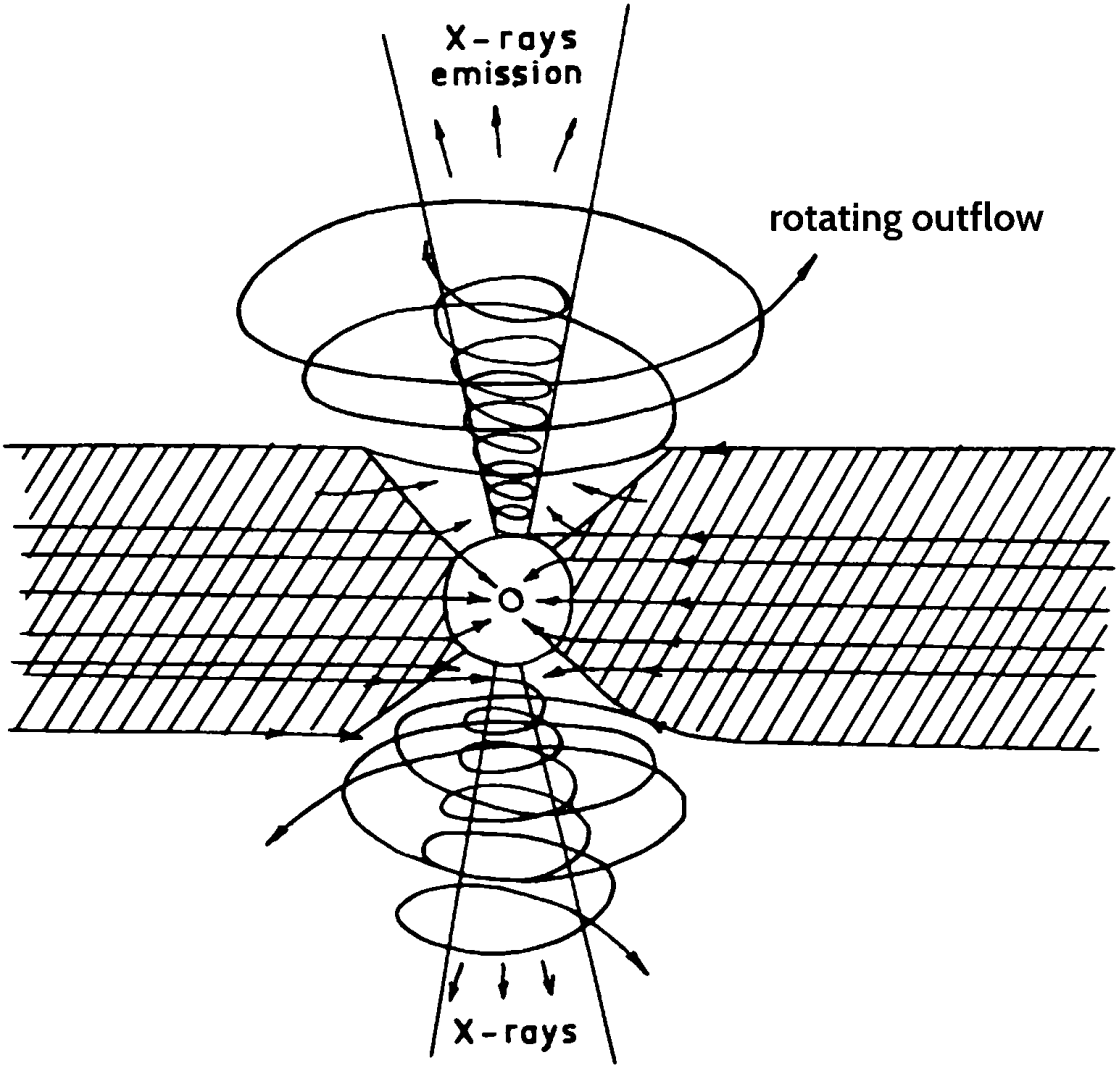
The outflow in the super-Eddington regime. Adapted from Shakura and Sunyaev (1973)

**Fig. 2:**
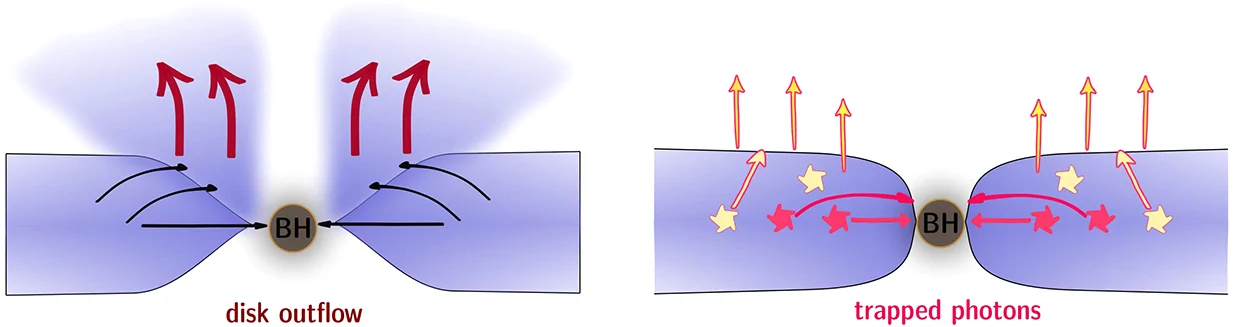
Schematic illustration of two important processes in the supercritical accretion flow towards a black hole: the disc outflow from the disc surface (left) and the photon trapping deep inside the disc (right). Adapted from Ohsuga and Mineshige (2014)

Shakura and Sunyaev (1973) in their work, which laid the foundation for studies of structure and emission of geometrically-thin accretion discs, also provided a scenario of the autoregulation mechanism for high accretion rates. According to their model, matter flows from the disc due to radiation pressure within the so-called spherisation radius (see Fig. 1). The outflow starts at the radius where the geometrically thin disc becomes sufficiently thick, namely, when the disc’s half-thickness

2 $$ z \sim \frac {3}{4} \,R_{\mathrm{g}} \, \frac{\dot{M} \, c^{2} }{L_{\mathrm{Edd}}} $$

becomes comparable to the radius itself (thus, the term spherisation radius $R_{\mathrm{sph}}$). If the accretion rate decreases linearly with radius inward of $R_{\mathrm{sph}}$, the local radiation pressure remains at a critical level up to the inner boundary of the disc. The spherisation radius is approximately equal to the photon trapping radius, as introduced by Begelman (1979), see Table 1. These two radii represent two primary effects that dictate the structure of discs in the supercritical regime: the outflow of mass from the disc surface and the effective capture of photons by the main flow spiraling toward the center, Fig. 2. Consequently, the luminosity does not increase proportionally with the accretion rate as in the standard thin disc; instead, it grows only logarithmically,

3 $$ L_{\mathrm{bol}} \approx L_{\mathrm{Edd}} \, (1+ \ln \dot{m}) \, , $$

where $\dot{m} = \eta \,\dot{M}\, c^{2} /L_{\mathrm{Edd}}$ and η is the accretion efficiency.

**Table 1:** Characteristic scales for super-critical accretion onto compact objects. Notations: Mass is scaled to solar units as $m = M/M_{\odot}$; the accretion rate is given in Eddington units as $\dot{m} = \dot{M}/\dot{M}_{\mathrm{Edd}}$, where $\dot{M}_{\mathrm{Edd}} = 1.39 \times 10^{18} \, m \text{ g s}^{-1}$. The disc aspect ratio is z/r. For a neutron star (NS) with polar magnetic field B, the magnetic dipole is $\mu = B R_{\mathrm{NS}}^{3}/2$, assuming a stellar radius $R_{\mathrm{NS}} = 10 \text{ km}$. For further details on the definitions of the spherisation and trapping radii, see Poutanen et al. (2007), King et al. (2023), and the relation of the magnetospheric radius on the accretion rate, Chashkina et al. (2019)

Characteristic Radius	Formula	Numerical Approximation
Disc inner radius around Schwarzschild BH	$3\, R_{\mathrm{g}} = \dfrac{6\,GM}{c^{2}}$	9 *m* km
Spherisation radius	$\dfrac{3}{8\pi} \dfrac{\dot{M}\sigma _{\mathrm{T}}}{c\, m_{\mathrm{p}}}$	22 *ṁ* *m* km
Trapping radius	$20\, \dfrac{z}{r}\, \dot{m} \,\dfrac{GM}{c^{2}}$	$30 \,\dfrac{z}{r}\,\dot{m}\, {m}\text{ km}$
Magnetospheric radius	$\sim 0.5 \left ( \dfrac{\mu ^{2}}{\dot{M}\,\sqrt{GM}} \right )^{2/7}$	$570 \, \dfrac{(B/10^{12}\, \mathrm{G})^{4/7}}{\dot{m}^{2/7}\, m^{{3/7}} } \text{ km}$

#### Advection and Wind

Towards the end of the 1970s, an elegant 3D analytic solution for the accretion with a high mass rate was developed. It was proposed that, because the photons can be trapped within the disc, a super-Eddington flow could exist without a wind, albeit with a constrained luminosity. This accretion flow, coined as a ‘Polish doughnut’, is a low-viscosity, optically thick and radiatively very inefficient, and exhibits the advection phenomenon, see the review by its founding father Abramowicz (2005).

The shape of the Polish doughnut is determined by the radial distribution of angular momentum and radiation pressure. If the disc torus possesses uniform angular momentum throughout, it will be notably thick and will appear from a distance as a spherical star-like object with a luminosity of approximately one Eddington. Its luminosity would display a pronounced angular dependence, with the majority of its emission concentrated in the direction of the central funnel. Regardless of a specific angular momentum distribution, the narrow funnel of such a torus could collimate jets and accelerate particles, as it was suggested for active galactic nuclei and Galactic source SS 433 by Lynden-Bell (1978), Sikora and Wilson (1981), Abramowicz and Sharp (1983), etc. It was shown later that strong advective cooling is actually tends to reduce significantly the geometrical thickness and luminosity of Polish doughnuts (Wielgus et al. 2016).

The local balance between the viscous heating and emission is not universally applicable even in the sub-Eddington accretion regimes, particularly in the regions where the disc thickness approaches its radius (due to proximity to the central object), z∼r. In 1981, Paczynski and Bisnovatyi-Kogan challenged the assumption of the local energy balance (also Hōshi and Shibazaki 1977) and proposed the energy equation accounted for the radial heat transfer, or advection. These discs were termed “slim”, even though the model suggested a relative thickness of up to 0.3.

In the case of super-Eddington accretion, when very thick discs are expected, advection is the inevitable cooling mechanism, related to the photon trapping. Abramowicz et al. (1988) developed the super-Eddington slim disc model in the Post-Newtonian potential and demonstrated the thermal stability of the new regime. The luminosity of the slim disc grows logarithmically with the accretion rate, similar to what is expected for super-Eddington outflow discs, Eq. (3). Advective cooling also ensures that the relative half-thickness of the slim disc z/r≲1 (see also Lasota et al. 2016).

At high accretion rates, the angular momentum transfer equation has to be modified as well, in order to take into account the radial pressure gradient and the inertial term, thus resulting in the appearance of a sonic point. Sądowski (2009) investigated the slim disc in the Kerr metric and obtained solutions that traversed the sonic point (see also Abramowicz et al. 1988; Artemova et al. 2001; Bisnovatyi-Kogan and Lovelace 2001). It was established that the solution for a given set of $\dot{M}$ and α is always unique and approaches at large radii the standard thin-disc solution obtained in the Newtonian limit.

Scenarios where outflows accompany advection of energy in discs have been proposed for the super-Eddington accretion as in SS 433 (Lipunova 1999, 1D model), and low accretion rates (ADIOS: advection-dominated inflow outflow solutions, Blandford and Begelman 1999). The latter is also applicable for high accretion rates in the form of adiabatic convective self-similar 2D solutions (Blandford and Begelman 2004). King and Begelman (1999) suggested that a radiation-pressure dominated version of ADIOS could explain the binary systems with almost all mass expelled during the mass-transfer, and suggested that mass loss occurs from the spherisation radius of a super-Eddington accretion disc. Dotan and Shaviv (2011) argued that inhomogeneities due to radiative hydrodynamic instabilities reduce the effective opacity and constructed the slim disc model, which can locally surpass the Eddington limit.

Assuming that the advection fraction remains constant, a 1D self-similar description of the advection-dominated optically thick accretion disc is possible (Watarai and Fukue 1999). For an advective disc without outflow, Watarai (2006) found an analytic form of $Q_{\mathrm{adv}}/Q_{\mathrm{vis}}$ for the polytropic EOS.

Watarai and Fukue (1999) calculated the radiation field of the slim disc and investigated the particles’ trajectories in the limit of an optically thin outflow, when the shape of the disc strongly influences the motion of particles. Kitabatake et al. (2002) included the power-law dependence of the accretion rate on the radius in the self-similar model and found that the thickness and surface temperature of discs with and without winds are similar.

**Fig. 3:**
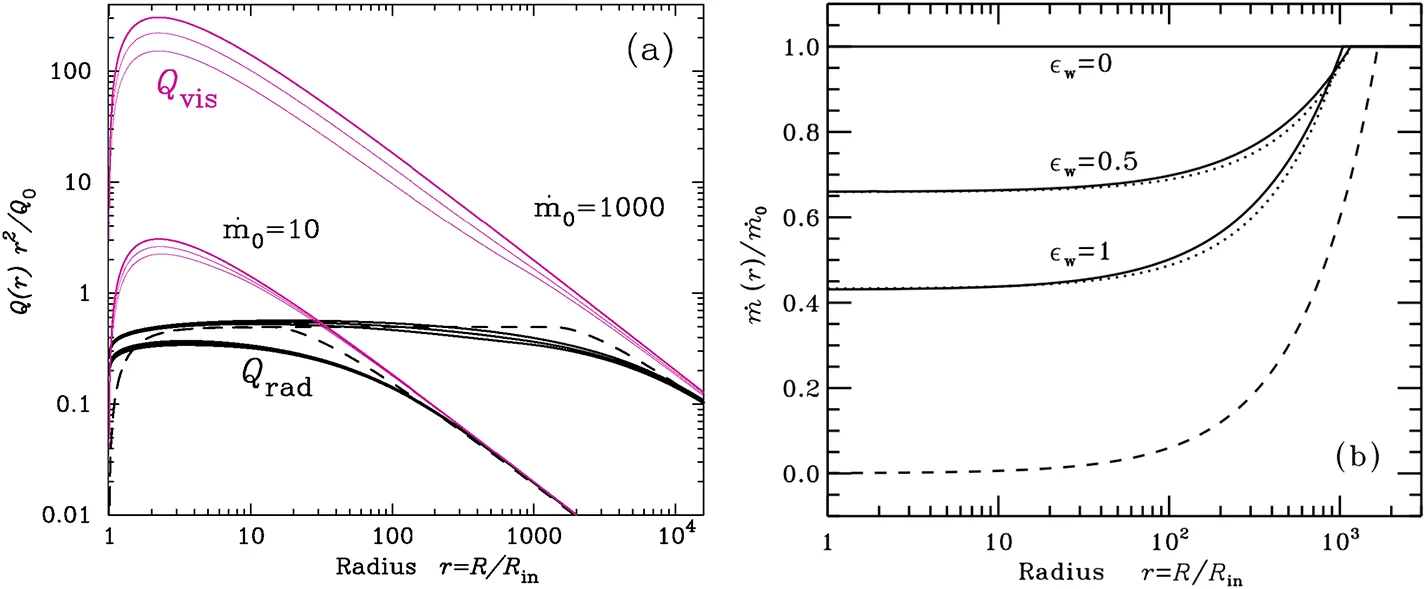
The viscous and radiative energy flux (a) and accretion rate (b) in the disc with outflow for $\dot{m}_{0}=10$ and $10^{3}$. The solid lines show the 1D model of an advective disc with different outflow efficiency $\epsilon _{\mathrm{w}}$, indicated in the right panel. The dashed lines represent the super-Eddington model of Shakura and Sunyaev (1973) without advection ($Q_{\mathrm{rad}} = Q_{\mathrm{vis}}$). The flux is measured in units of $Q_{0} = c^{5} \, m_{p}/ (36 G M \sigma _{\mathrm{T}})$. The dotted curves in (b) are analytic approximations by Poutanen et al. (2007). Most mass loss occurs close to the spherisation radius. Figure adapted from Poutanen et al. (2007)

The structure and observational appearance of optically thick outflows launched from the super-Eddington advection-dominated disc was considered by Poutanen et al. (2007). The mass-loss rate in the wind was found from the law of conservation of energy: a fixed fraction $\epsilon _{\mathrm{w}}$ of the radiative flux is assumed to go to the kinetic energy of the outflow, Fig. 3. In the case of $\epsilon _{\mathrm{w}}=1$, this ansatz yields the same linear dependence of $\dot{M}$ in the disc (Lipunova 1999), as originally proposed in the scenario of Shakura and Sunyaev. Since the vertical structure of such 1D disc is treated approximately, the emitted flux, which is the difference between the generated and advected heat, and the outflow mass rate depend also on the way the vertical structure equations are integrated.

In the case of super-Eddington accretion onto a magnetized neutron star, within the framework of auto-regulation by the wind, the maximum accretion rate onto the star depends on the star’s magnetic dipole moment, $\dot{M} \sim \mu ^{4/9}\, L_{\mathrm{Edd}}^{7/9} \, (GM)^{-8/9} $ (Lipunov 1982). This is because, in such a regime, the inner radius of the disc is a function of the magnetic moment and mass only, $R_{\mathrm{in}} \propto \mu ^{4/9} M^{-1/9}$, and is limited from below when the accretion rate increases. Chashkina et al. (2019) constructed a 1D model of a slim disc around a neutron star and found that, while accounting for the wind leads to an increasing inner disc radius, the scaling of the magnetospheric radius is similar to the classical Alfven relation (see Table 1) when the disc becomes advection-dominated (i.e. the accretion stopping radius is sensitive to the accretion rate).

### Predicted Observational Characteristics

The super-Eddington advection-dominated disc is radiatively inefficient due to the long radiative diffusion time. It is therefore no coincidence that its dynamical properties are similar to those of another advection-dominated solution that is realised for very low accretion rates (ADAF), but due to the long cooling time. The effective temperature of super-Eddington advection-dominated disc depends on radius more weakly than in the standard geometrically thin disc, as $T_{\mathrm{eff}} \propto r ^{-1/2}$ (Wang and Zhou 1999; Watarai and Fukue 1999). While the density profile in the super-Eddington disc is modified if there is an outflow,^1^ the effective temperature has the same radial dependence and the disc thickness is still proportional to the radius (Kitabatake et al. 2002; Wu et al. 2002).

**Fig. 4:**
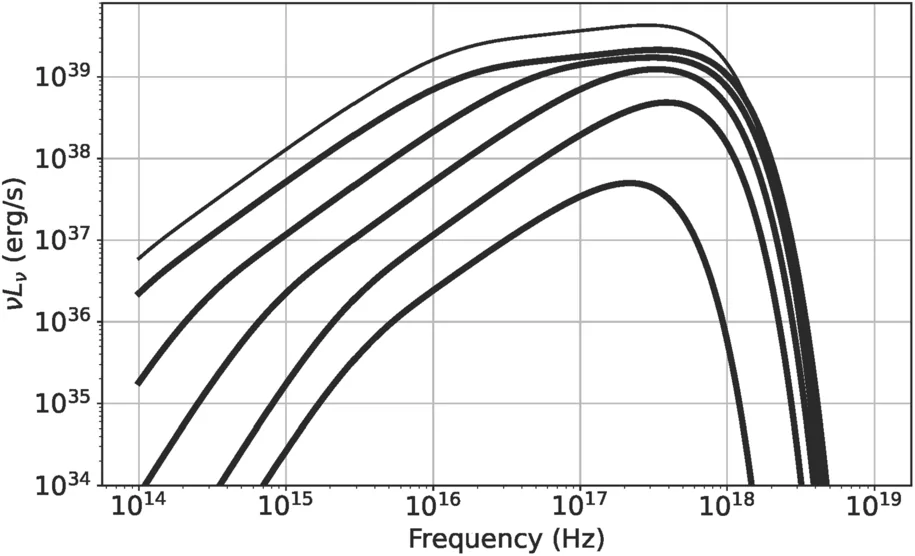
Emergent spectrum from the accretion disc around $10\, M_{\odot}$ black hole (thick lines) with accretion rates from $10^{3}$ to $0.1 \times \dot{M}_{\mathrm{Edd}}$, from top to bottom, where the Eddington accretion rate $\dot{M}_{\mathrm{Edd}} = 1.39 \times 10^{19} (M/10 M_{\odot})\text{ g}/\mathrm{s}$. The Eddington luminosity $L_{\mathrm{Edd}} = 1.25 \times 10^{39}\,(M/10 M_{\odot}) $ erg/s. The thin line shows the spectrum of the slim disc with around $20\, M_{\odot}$ black hole with $\dot{M}=10^{3} \dot{M}_{\mathrm{Edd}}$

Advection has a characteristic imprint on the disc spectrum, — it produces a flattened interval in the SED, see Fig. 4. Wang et al. (1999) found that the maximum characteristic energy of the photons does not depend on the accretion rate, but depends on the BH mass (see also Mineshige et al. (2000), Watarai et al. (2000)). Kitabatake et al. (2002), Fukue (2004) investigated supercritical accretion discs with winds, wherein the accretion rate is a power-law function of the radius, and found that the disc thickness and effective temperature were similar to those observed in a supercritical disc without winds.

The above results indicate that the observational characteristics of supercritical discs, whether with or without winds, would not differ significantly, except in cases where the wind is not optically thin. Poutanen et al. (2007) investigated observed characteristics of a source with supercritical accretion and an optically-thick wind. The temperatures at characteristic radii were determined, including the outer photosphere of the wind, the spherisation radius, and the innermost edge of the disc. It was suggested that ULXs are super-Eddington accretion discs in binary systems observed close to normal to the disc symmetry plane.

The intense accretion disc wind might block the emitted X-rays from most of the viewing angles and leave only narrow optically-thin funnel along the disc’s rotational axis. Geometrical collimation, e.g. by scattering-off inner outflow walls, should result in the beaming of emission, which would make observed isotropic luminosities of super-Eddington discs higher than intrinsic $L_{\mathrm{bol}}$ (King 2009). This should have important consequences for the observed characteristics, see review on observational properties and theory for Ultraluminous X-ray Sources by King et al. (2023).

Thick accretion discs with vortexes, where the radiation pressure accelerates matter to relativistic velocities, were theorised to collimate radiation and accelerate plasma by Lynden-Bell (1978), Abramowicz and Piran (1980). Mildly relativistic velocities may be attained, limited by the effect of the radiation drag (Phinney 1982; Fukue 2024).

In the case of super-Eddington accretion onto a neutron star, most of the energy is radiated close to the surface of the neutron star, unless powerful feedback mechanisms prevent the mass from reaching the surface (Zel’dovich and Shakura 1969). For a magnetized neutron star, the channeling of the accretion flow along magnetic tubes, combined with the reduction of scattering cross-sections in the strong magnetic field, can accommodate high accretion rates and produce luminosities exceeding the Eddington limit (Mushtukov et al. 2015). At such rates, radiative shocks were predicted to form above the star’s surface (Davidson and Ostriker 1973; Inoue 1975; Basko and Sunyaev 1976). The time-dependent 1D model of Abolmasov and Lipunova (2023) reproduces the shock position and flow dynamics and also predicts mildly relativistic outflows from the magnetosphere at high accretion rates. The structure of the magnetospheric flow is critical for its observational characteristics (Mushtukov et al. 2017). These characteristics are also strongly influenced by the complex picture of photon propagation in strong magnetic and gravitational fields, which is currently being investigated using numerical methods.

### Numerical Approaches

#### Super-Eddington Accretion onto Black Holes

As described in the preceding sections, the structure of super-Eddington accretion disks has been extensively discussed based on a variety of theoretical models, with the slim disk model being among the most representative. To determine a more realistic structure, multi-dimensional radiation hydrodynamic (RHD) simulations have been conducted. These simulations numerically solve the coupled equations of hydrodynamics, radiative transfer, and radiation-gas interactions, and are essential for understanding super-Eddington disks dominated by radiation pressure and the associated radiatively driven outflows. While the transport of angular momentum and the dissipation of energy in the disk are known to be of magnetic origin, necessitating radiation magnetohydrodynamic (RMHD) simulations for greater accuracy, these aspects will be discussed later.

**Fig. 5:**
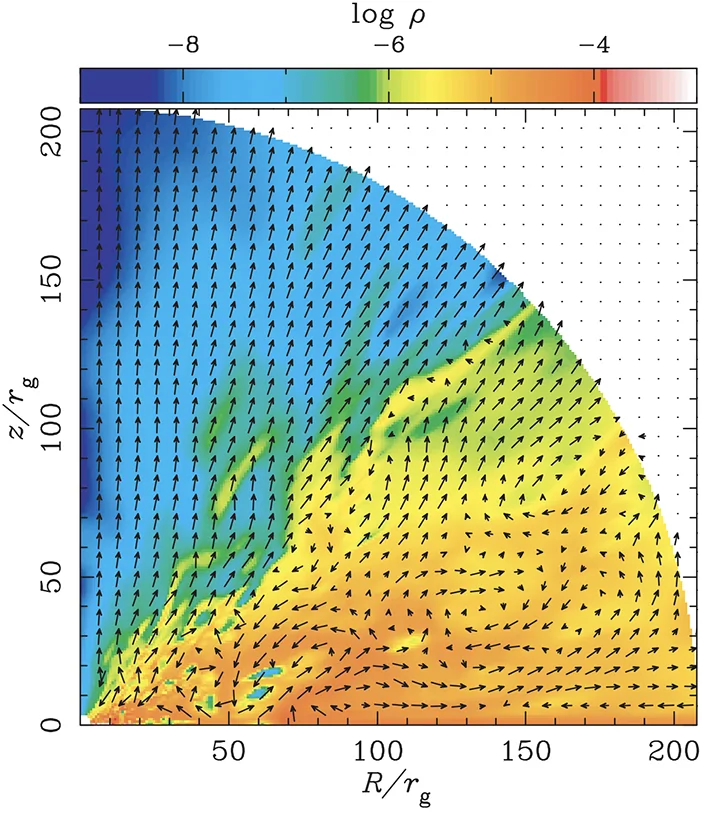
Snapshot of the super-Eddington accretion disk around the black hole from the RHD simulation. Color contours show gas density; arrows denote velocity. A high-density, geometrically thick disk (orange and yellow) forms near the equatorial plane, while an outflow (light blue) emerges above it Ohsuga et al. (2005)

A pioneering study that demonstrated the self-consistent formation of a super-Eddington disk and its outflows, without assuming an initial disk configuration, was conducted by Ohsuga et al. (2005) (see also Eggum et al. 1988; Okuda and Fujita 2000). Their simulations revealed the formation of a geometrically and optically thick, radiation-pressure-dominated disk, where a large number of photons are advected into the black hole along with gas due to photon trapping (Fig. 5) (see also Ohsuga and Mineshige 2007). Radiatively driven outflows are launched from the surface of the disk, and the emitted radiation flux is mildly collimated along the rotational axis. As a result, when such a system is observed face-on, it can appear significantly brighter than its intrinsic luminosity. Kitaki et al. (2018) reported that the radial density profile of the super-Eddington disk reproduced by RHD simulations is somewhat flatter than the $r^{-1.5}$ profile predicted by the slim disk model, instead following approximately $r^{-0.73}$. This flattening is likely attributable to convective motions.

Although the RHD simulations mentioned above adopted an alpha-viscosity prescription, the origin of viscosity in accretion disks had already been established as magnetic turbulence (Balbus and Hawley 1991). Accordingly, subsequent efforts removed the alpha-viscosity approximation and incorporated magnetic effects by conducting radiation magnetohydrodynamic (RMHD) simulations of super-Eddington accretion flows (Ohsuga et al. 2009; Ohsuga and Mineshige 2011). These simulations reaffirmed the presence of geometrically and optically thick, radiation-pressure-dominated disks, as demonstrated in the RHD studies, and further elucidated the formation of radiatively accelerated and magnetically collimated jets. It has recently been suggested that gas pressure from disk winds may be necessary for collimation. In principle, RMHD simulations are capable of reproducing not only super-Eddington disks, but also radiatively inefficient accretion flows (RIAFs) and standard thin disks. However, simulations of standard disks require significantly higher resolution due to their geometrical thinness.

**Fig. 6:**
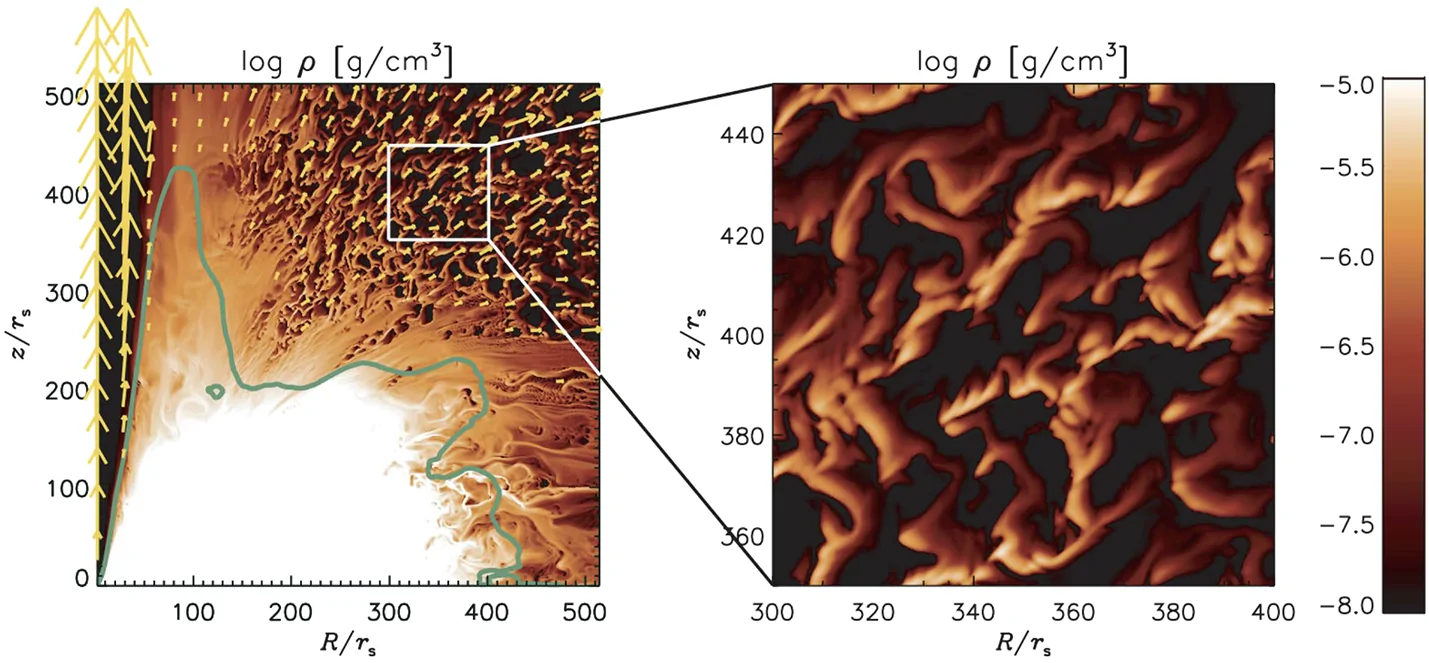
The clumpy wind obtained from the RHD simulation. Color contours show the gas density, and arrows denote the velocity. The outflow launched from the super-Eddington disk (the white region near the equatorial plane) fragments into numerous gas clouds. The right panel shows a magnified view. Takeuchi et al. (2013)

High-resolution, large-domain RMHD simulations have revealed that disk winds fragment into numerous gas clouds (see Fig. 6; Takeuchi et al. 2013; Kobayashi et al. 2018). This fragmentation is attributed to Rayleigh-Taylor instabilities in radiation-pressure-dominated regions, and the resulting clumpy winds may be responsible for the observed X-ray variability in super-Eddington sources, including ULXs (Middleton et al. 2011). Recent observations by the XRISM satellite have identified multiple absorption lines associated with ultra-fast outflows (UFOs), and their potential connection to clumpy winds has attracted considerable attention (XRISM collaboration 2025).

Initial RMHD simulations were based on semi-relativistic formulations, but have since evolved into fully special relativistic (SR) and general relativistic (GR) frameworks. SR-RMHD simulations have shown that radiation drag suppresses the terminal velocity of radiatively driven jets (Takahashi and Ohsuga 2015). The resulting jet speeds typically reach a few to several tens of percent of the speed of light, consistent with observations of SS433 and blue-shifted spectral lines in ULXs (Margon 1984; Pinto et al. 2016, 2017b; Kosec et al. 2018).

GR-RMHD simulations of super-Eddington accretion were pioneered by McKinney et al. (2014) and Sądowski et al. (2014). These simulations confirmed the emergence of geometrically and optically thick, radiation-pressure-dominated disks, consistent with previous results (see also Takahashi et al. 2016; Thomsen et al. 2022a; Curd and Narayan 2023). Lasota et al. (2016) reported that, in the case of zero black hole spin, the scale height of the GR-RMHD disk increases with accretion rate, eventually reaching a constant value of approximately 0.6−0.7 in the high accretion regime. This is largely consistent with the slim disk model. Additionally, the surface density-accretion rate relation obtained from GR-RMHD simulations was found to agree with that predicted by the slim branch of disk theory.

**Fig. 7:**
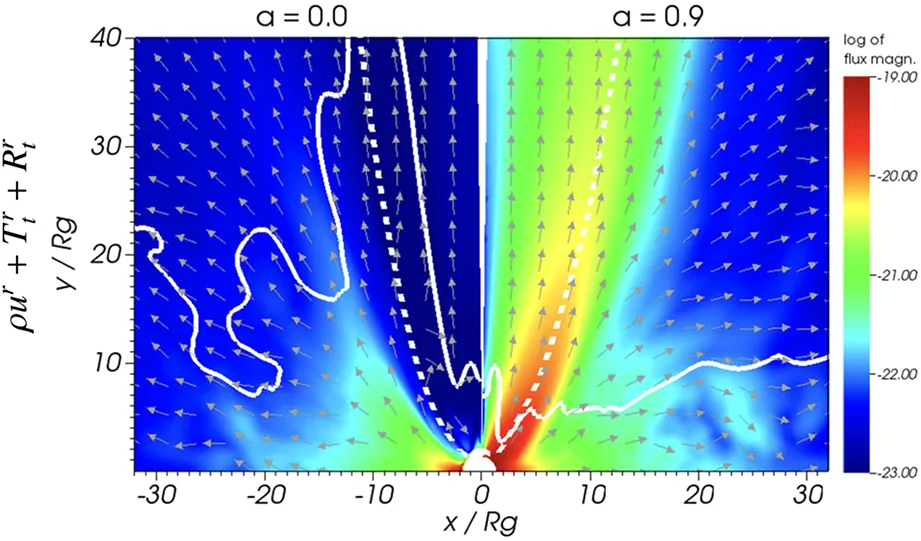
Energy flux distribution (total energy minus rest mass) obtained from the GR-RMHD simulation. The case with a spin parameter of 0.9 (right panel) exhibits a stronger energy flux than the non-spinning case (left panel). Sądowski et al. (2014)

**Fig. 8:**
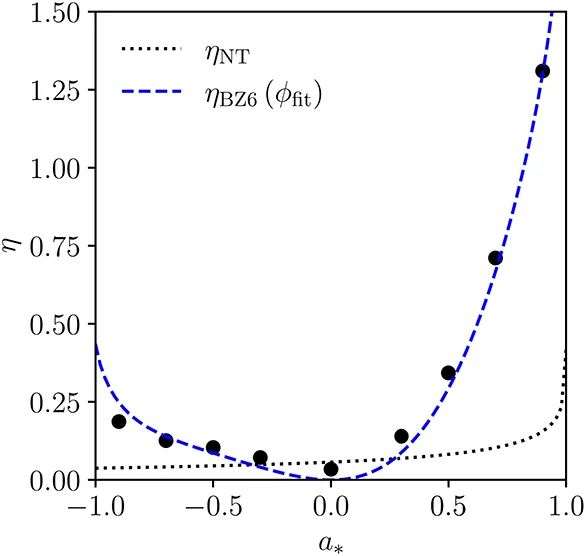
Energy conversion efficiency of a super-Eddington accretion disk in the MAD state obtained from GR-RMHD simulations. The resulting efficiency (black circle) is higher when the black hole is spinning, in rough agreement with the spin dependence expected from the Blandford-Znajek (BZ) mechanism (blue-dashed line) Narayan et al. (2022)

One of the key insights from GR-RMHD simulations is that black hole spin enhances the energy conversion efficiency and leads to powerful jet formation. For example, while the efficiency is about 5% for non-spinning black holes, it increases to 33% for spin parameter a=0.9 (see Fig. 7; Sądowski et al. 2014). Narayan et al. (2022) showed that, in the magnetically arrested disk (MAD) regime, the efficiency can reach up to 140% (see Fig. 8). Such an enhancement in efficiency is thought to be driven by the Blandford-Znajek (BZ) mechanism (Blandford and Znajek 1977). Furthermore, the jet opening angle in MAD states increases with both the black hole spin and the accretion rate (Narayan et al. 2022; Curd and Narayan 2023). Intermediate states between MAD and SANE, as well as pure SANE conditions, have also been explored (Utsumi et al. 2022), revealing that higher spins yield greater efficiency, particularly in terms of energy release via Poynting flux. Specifically, the dominant energy output mechanism shifts from radiation at a=0 to magnetic energy at a=0.9. Given that the ratio $L_{\mathrm{kin}} / L_{\mathrm{rad}}$ (where $L_{\mathrm{kin}} $ and $L_{\mathrm{rad}} $) depends on spin, ULXs with high ratios (e.g., IC 342 X-1, $L_{\mathrm{kin}} / L_{\mathrm{rad}} \sim 0.15$-0.3) may host spinning black holes, while those with lower ratios (e.g., Holmberg II X-1, $L_{\mathrm{kin}} / L_{\mathrm{rad}} \sim 0.04$-0.14) may host non-spinning ones. However, variations in this ratio could also reflect differences in the accretion rate (Kitaki et al. 2021).

The long-term evolution of the black hole spin parameter under GR-RMHD simulations has also been discussed. As the spin energy is extracted via the BZ mechanism, the angular momentum of spinning black holes decreases. This spin-down effect is stronger for higher spin values, suggesting that MAD-state super-Eddington disks evolve toward a spin parameter close to zero (Narayan et al. 2022; Ricarte et al. 2023). However, it remains unclear whether the MAD state can be sustained over long durations, as some simulations show a transition from MAD to SANE configurations (Curd and Narayan 2023).

In addition to the BZ mechanism, the Lense-Thirring (LT) precession, another phenomenon arising from black hole spin is of great interest. LT precession may explain the periodic change in the direction of the jet (Cui et al. 2023) and is also considered a possible origin of quasi-periodic oscillations (QPOs) (Ingram and Motta 2019). Although many studies have employed GR-MHD simulations to investigate this effect (Fragile et al. 2007; Liska et al. 2018, 2019), recent GR-RMHD simulations have begun to explore LT precession in super-Eddington disks (Asahina and Ohsuga 2024). Future studies should clarify the connection between such precession and observational features such as QPOs in ULXs (Atapin et al. 2019) or the time-variable jet direction in V404 Cyg (Miller-Jones et al. 2019). The relation between LT precession and QPEs (quasi-periodic eruptions) is also of interest. Middleton et al. (2025) suggest that QPEs may result from precessing super-Eddington accretion disks viewed at high inclination angles, based on the radiation properties obtained from GR-RMHD simulations.

Most GR-RMHD simulations to date have focused on the regions close to the black hole, typically well within the photon trapping or spherization radius. This limitation arises because the viscous timescale increases with distance from the black hole, making long-term simulations computationally expensive. To investigate the global structure of super-Eddington disks, large-domain, long-duration simulations have been developed. Although these studies often employ semi-relativistic RHD simulations with an alpha-viscosity model, they have shown that outflows are launched from the entire region within the trapping radius (Kitaki et al. 2021; Yoshioka et al. 2022; Hu et al. 2022). Interestingly, the highest mass outflow rates are not seen near the black hole, but somewhat further out (which matches predictions from some analytical models: see Sect. 2.1). Moreover, near the trapping radius, failed outflows that do not reach escape velocity and eventually return to the disk have been identified. When applied to ULXs, the outflow power derived from these large-scale simulations is in the range of $10^{39}-10^{41} \mathrm{erg\,s^{-1}}$, consistent with the energetics of observed ULX bubbles (Hashizume et al. 2015; Kitaki et al. 2021; Yoshioka et al. 2022). Sądowski and Narayan (2015) further suggest that, as the outflows propagate away from the black hole, their radiative energy is gradually converted into kinetic energy. Verification of this hypothesis through large-domain simulations remains an important future challenge.

**Fig. 9:**
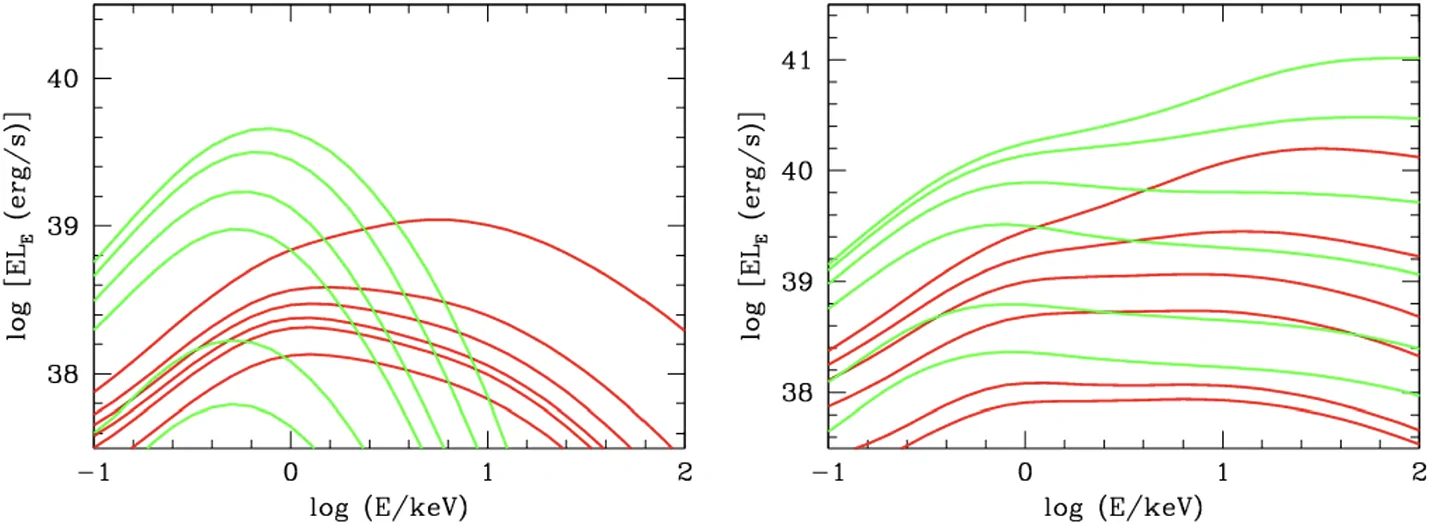
Spectra of super-Eddington accretion disks in the MAD state. Left panel: spectra for a non-spinning black hole with mass accretion rates of 13 (green) and 230 (red) times the Eddington limit. Right panel: spectra for a spinning black hole with a spin parameter of 0.9 and mass accretion rates of 68 (green) and 360 (red) times the Eddington limit. The spectra become harder with increasing accretion rate and spin parameter Takahashi and Ohsuga (2017)

Super-Eddington accretion disks launch jets along the rotational axis, accompanied by surrounding disk winds. Due to Compton scattering occurring within the jets and winds, the emergent radiation spectrum deviates from a multicolor disk blackbody. Kawashima et al. (2012) computed the spectra of super-Eddington flows based on RHD simulation results using post-processing radiation transfer calculations. They found that the spectral energy distribution (SED) strongly depends on the mass accretion rate and observer’s viewing angle, and successfully reproduced the observed SEDs of ULXs (see also Kitaki et al. 2017). Narayan et al. (2017) later showed, based on GR-RMHD simulations, that the SED of a super-Eddington disk around a spinning black hole is significantly harder than that around a non-spinning black hole (see Fig. 9). Pacucci and Narayan (2024) applied GR-RMHD-based SEDs to Little Red Dots (LRDs), suggesting that the absence of strong X-ray emission in most LRDs can be explained by a moderately super-Eddington disk (with an Eddington ratio of 1.4-4) around a non-spinning black hole, observed at an inclination angle above 30 degrees. However, accurately resolving the high-temperature, low-density polar region remains challenging in hydrodynamics simulations. Developing improved methods for comparing theoretical predictions with observed high-energy radiation remains a key issue. Gu et al. (2016) and Ogawa et al. (2017) showed that edge-on observations can obscure high-energy emission, leading to a softened SED. This result supports the hypothesis that ultraluminous supersoft sources (ULSs) may be ULXs viewed at high inclinations (Urquhart and Soria 2016).

#### Super-Eddington Accretion onto Neutron Stars

**Fig. 10:**
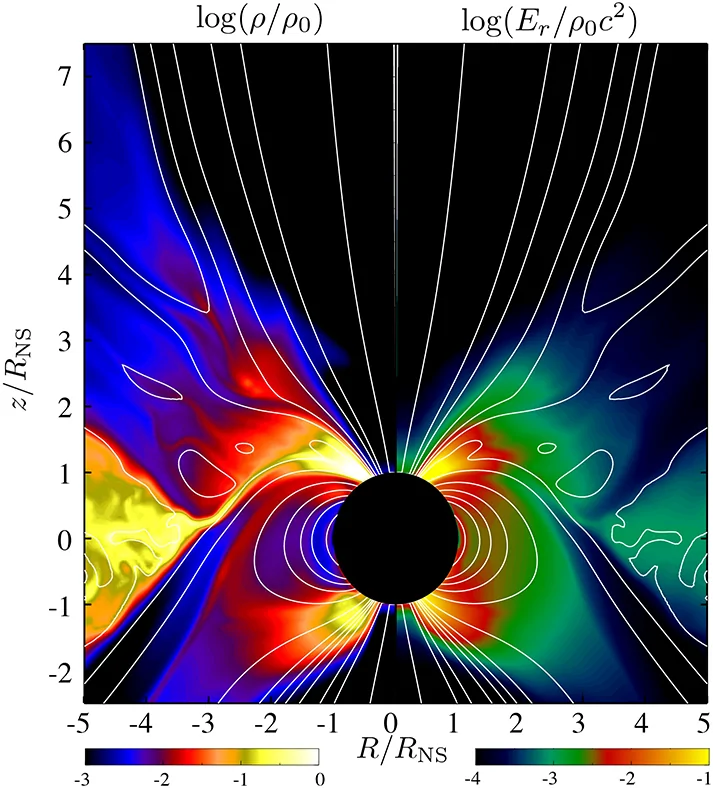
Structure of super-Eddington accretion onto a neutron star with a dipole magnetic field, obtained from GR-RMHD simulations. The left panel shows the gas density, and the right panel shows the radiation energy density. Accretion columns form as gas accretes along the magnetic axis. © H. R. Takahashi

RHD simulations of super-Eddington disk accretion onto non-magnetized neutron stars were initially performed by Ohsuga (2007), and were later extended to GR-RMHD frameworks (Takahashi et al. 2018; Abarca et al. 2018). As in the case of BHs, these simulations revealed the formation of geometrically and optically thick disks accompanied by radiation-driven outflows. However, a key difference is the appearance of a high-density region in the immediate vicinity of the NS surface, which is attributed to the presence of a solid surface. Super-Eddington accretion onto magnetized NSs with dipolar magnetic fields was investigated by Takahashi and Ohsuga (2017) (see also Fig 10, Abarca et al. 2021; Inoue et al. 2023). In this case, a magnetosphere forms around the NS, and the disk is truncated at the magnetospheric radius. Gas flows along magnetic field lines and accretes onto the magnetic poles, forming accretion columns. This picture is consistent with the accretion curtain model proposed by Mushtukov et al. (2017). Because weaker dipolar fields allow more efficient radiative beaming, it has been suggested that ULX pulsars may host weakly magnetized NSs (Kayanikhoo et al. 2025). Since there is no guarantee that the dipolar field dominates the magnetic structure, GR-RMHD simulations incorporating quadrupolar magnetic fields have also been performed (Inoue et al. 2024), showing that when the quadrupole component dominates, equatorial accretion belts can coexist with polar accretion columns. Most studies assume alignment between the magnetic and rotation axes to simplify the problem, but relaxing this assumption is likely crucial for interpreting the pulse profiles of ULX pulsars. While still within the scope of GR-MHD, simulations with misaligned magnetic axes have been carried out (Das and Porth 2024; Murguia-Berthier et al. 2024).

The structure of super-Eddington accretion columns that form near the magnetic poles has been investigated using RHD simulations (Kawashima et al. 2016; Kawashima and Ohsuga 2020). These studies revealed that shock appears within the accretion column, and the large amount of radiation energy generated is released through the side surface of the column. As a result, the emission from the accretion column is not pencil-beamed but rather fan-beamed. RMHD simulations have also been performed (Zhang et al. 2022), demonstrating broadly similar internal structures as in the RHD simulations. Notably, however, these RMHD simulations newly reported the emergence of quasi-periodic luminosity oscillations in the 2–10 kHz range.

Accounting for the influence of the photon energy, resonances, and polarization modes on the Compton scattering cross-section has shown that in strong magnetic fields, the effective scattering cross-section is reduced, allowing for super-Eddington luminosities exceeding the canonical limit (Mushtukov et al. 2015). In ultra-strong magnetic fields such as those of magnetars ($>10^{14}~\mathrm{G}$), luminosities can potentially exceed $10^{40}~\mathrm{erg~s^{-1}} $. RMHD simulations that explicitly incorporate the magnetic suppression of the scattering cross-section have also been carried out (Zhang et al. 2023).

In the case of strongly magnetized neutron stars, anisotropic opacity can arise, and numerical instabilities associated with low plasma beta are more likely to occur, making simulations increasingly challenging. In addition, the expansion of the magnetosphere increases the required computational domain, which further raises the computational cost. As a result, the numerical exploration of super-critical accretion onto very strongly magnetised neutron stars is currently limited but remains an important frontier.

In simulations of super-critical accretion onto neutron stars, boundary conditions have been adopted in which gas is absorbed at the stellar surface while radiation is reflected (e.g., Ohsuga 2007; Kawashima and Ohsuga 2020), as well as conditions in which both gas and radiation are reflected (e.g., Takahashi and Ohsuga 2017; Inoue et al. 2023). Although no study has clearly compared these different treatments, it is highly likely that the results depend on the adopted boundary condition.

Several numerical studies have investigated the radiation spectra of super-Eddington accretor around NSs. For instance, multi-temperature blackbody emission based on the accretion curtain model has been explored (Mushtukov et al. 2017; Koliopanos et al. 2017). Conforti et al. (2025) adopted a simple setup in which a magnetized NS ($10^{12}$–$10^{13}~\mathrm{G}$) is embedded in a torus-shaped accretion flow, and examined models to reproduce observations of M51 ULX-7 and NGC 7793 P13. GR-RMHD simulations by Inoue et al. (2023) demonstrated that optically thick outflows accelerated by radiation pressure can account for the blackbody emission observed from Swift J0243.6+6124 (Tao et al. 2019). Mushtukov et al. (2018) calculated the pulse profiles of ULX pulsars (ULXPs), taking into account both direct emission from the accretion column and reflection from the NS surface. Their results showed that the observed flux can be either amplified by gravitational lensing or attenuated by eclipses caused by the companion star. Inoue et al. (2020) computed the pulse profiles produced by accretion columns and found that the pulsed fraction (PF) can be less than 50%, consistent with the observations of many ULXPs. Mushtukov et al. (2021) conducted Monte Carlo radiative transfer simulations to investigate the PF from NSs partially enclosed by the inner wall of a geometrically thick disk. They found that the PF is reduced when geometrical beaming is effective, and increases when beaming is absent. Based on this result, and the observational fact that ULXPs with PFs below 10% are rare, they inferred that most ULXPs are not subject to strong geometrical beaming.

#### Future Work

With advancements in numerical techniques, increasingly realistic and dynamic models of super-Eddington accretion disks have been realized. However, several important issues remain unresolved. This section outlines some of the outstanding challenges.

One major area for improvement lies in the treatment of the radiation field. In semi-relativistic RHD/RMHD simulations, the flux-limited diffusion (FLD) approximation (Levermore and Pomraning 1981) has been widely used, while more recent special and general relativistic simulations employ the M1 closure method (González et al. 2007), which provides slightly higher accuracy than FLD. The M1 closure is also more suitable for large-scale parallel computations, as it solves hyperbolic equations. However, the M1 closure is also an approximate method and it is known to fail in optically thin, highly anisotropic radiation fields (Asahina et al. 2020). GR-RMHD simulation codes that directly solve the radiative transfer equation, and capable of achieving higher accuracy than approximate methods such as FLD and M1, are currently under development (Asahina and Ohsuga 2022; White et al. 2023). Although non-relativistic, some RMHD simulations that solve radiative transfer directly have already been used to study super-Eddington disks (Jiang et al. 2014, 2019).

Another possible requirement is the implementation of two-temperature simulations that distinguish between ion and electron temperatures. Although super-Eddington disks are generally dense enough that ion and electron temperatures are expected to equilibrate, the low density and high velocity may invalidate the single-temperature approximation in the jet region near the rotation axis. Two-temperature GR-RMHD simulations have been performed for radiatively inefficient accretion flows (Sądowski et al. 2017; Ryan et al. 2018); however, their application to super-Eddington accretion remains an important next step. Furthermore, in highly magnetized regions near the polar axis, accurate simulations often become difficult, for example due to the gas pressure becoming negative. To stabilize the computations, techniques such as applying a numerical floor have been employed; however, the introduction of transitions to a force-free regime has also been proposed in GR-MHD contexts (Parfrey and Tchekhovskoy 2017).

Large-domain GR-RMHD simulations remain a critical challenge. Within the photon trapping radius, both radiation trapping and outflow launching occur. Thus, simulations must extend from the black hole vicinity to the trapping radius to fully capture the disk structure. More precisely, long-duration simulations are needed to o extend the region where the net accretion rate is constant out to the trapping radius. While this condition is being gradually achieved in RHD simulations (Kitaki et al. 2021; Yoshioka et al. 2022; Hu et al. 2022), such simulations have not yet been realized within GR-RMHD frameworks (One of the most advanced studies is Fragile et al. (2025)). Even more ambitiously, simulations covering scales from the black hole to galactic dimensions are ultimately required. MHD-based attempts have been made (Cho et al. 2024; Guo et al. 2024), but GR-RMHD modeling of super-Eddington accretion across these scales remains an open problem.

Another unresolved issue is the formation of clumpy winds in super-Eddington accretion. Recent observations by XRISM have revealed outflows with mass-loss rates far exceeding the Eddington rate and exhibiting clumpy structures (XRISM collaboration 2025). Earlier simulations by Takeuchi et al. (2013) and Kobayashi et al. (2018) demonstrated the fragmentation of radiatively driven disk winds into gas clouds, but more detailed studies using high-precision methods are needed. It is also necessary to perform line-resolved radiative transfer calculations to produce synthetic X-ray spectra that can be directly compared with observation data by XRISM. QPOs observed in ULXs also present a significant problem that super-Eddington simulations must address. LT precession is one proposed origin for both long and short timescales QPOs (Middleton et al. 2018, 2019, and while GR-RMHD simulations of such precession have been performed (Asahina and Ohsuga 2024), their durations remain limited. To conduct power spectral analyses and assess sensitivity to initial conditions, longer GR-RMHD simulations are required. Another possible QPO production model is that of Okuda et al. (2009), who suggest that disc thermal instabilities can develop, leading to recurrent hot blobs with variable sizes rising through the accretion disc and the polar funnel region, producing 40-100 mHz QPOs. These models differ completely in their physical origin, and there are even more possibilities should one consider the role of the surface and strong magnetic field in the case where the compact object is a neutron star (see for instance Mushtukov et al. 2024); clearly there is a great deal of numerical work needed to address the origin of quasi-coherent timing features in super-critical discs.

Transient super-Eddington accretion episodes also deserve further attention. Such episodes may occur during tidal disruption events (TDEs) and quasi-periodic eruptions (QPEs). Simulations are needed to explore the fluid behavior of gas during these brief supercritical phases, as well as radiative transfer calculations to interpret their observational signatures.

In summary, while remarkable progress has been made in modeling super-Eddington accretion disks through advanced numerical simulations, a number of key challenges remain. Addressing these issues will be crucial for achieving a comprehensive theoretical understanding that aligns with the growing wealth of observational data.

## Observations of Supercritical Discs

The main observational indicators of super-Eddington accretion, as summarized above, are radiatively driven outflows and collimation/anisotropy. The role of radial advection within this picture is important, although the total accretion luminosity and emergent spectrum for either a slim disc or a classic SS73 supercritical disc is somewhat agnostic (Poutanen et al. 2007). Below we discuss in brief the evidence, either in support, or in tension with the theoretical work carried out to-date (for a more detailed discussion see King et al. 2023).

### X-ray Spectra

**Fig. 11:**
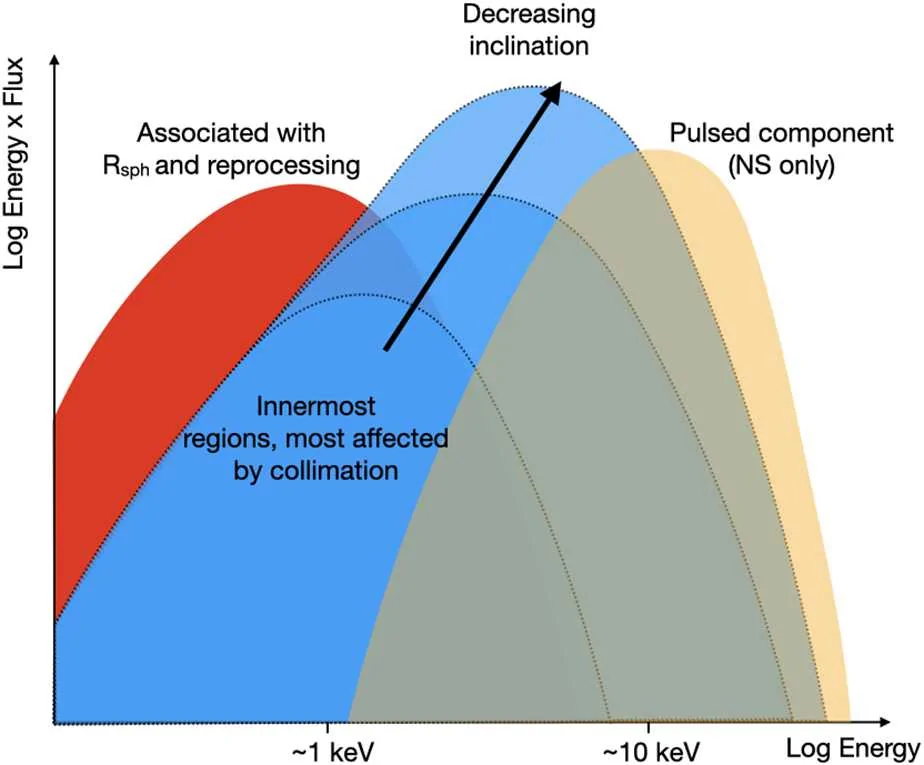
Example composite spectra from a super-Eddington source with emission from the outer super-Eddington disc/wind in red (from around R_sph_) which contains some component due to reprocessing of inner disc emission. At smaller radii we expect larger collimation effects as shown by the changing strength of the blue component. In gold is included an example pulsed component seen only in some neutron star ULXs (see e.g. Walton et al. 2018b; Brightman et al. 2016, and the review of King et al. 2023)

As illustrated in Fig. 11, the broadband (typically 0.3-30 keV) X-ray spectra of ULXs (noting that substantial energy may also be emitted at lower energies, e.g. Kaaret et al. 2010), can typically be deconvolved into at least two components by applying spectral-timing methods (Middleton et al. 2015, 2019), with neutron star systems indicating a third component associated with pulsed emission (Walton et al. 2018a; Brightman et al. 2016). Disregarding the latter, the spectral components may be identified with the radial regions suggested by Poutanen et al. (2007), with the softest emission being that from an outflow-modified disc peaking around the spherisation radius (flat in EfE modified by some beaming, depending on accretion rate). The expectation is that such a component should not show an L ∝ T^4^ relationship as would a standard thin accretion disc, and indeed, this would seem to be the case (Kajava and Poutanen 2009; Walton et al. 2020). Conversely, the harder spectral component is expected to be relatively insensitive to accretion rate (Poutanen et al. 2007, and in the case of neutron stars, have a weak relationship due to the roles of irradiation and advection: Chashkina et al. 2019). Spectral fitting suggests a relation that may be consistent with a fixed inner radius for this component (e.g. Walton et al. 2020, 2025 although there may yet be degeneracies in the spectrum).

The presence of outflows (see next section) leads to a geometrical unification model where the inner regions are collimated and only visible at smaller inclinations. The effect on the spectrum is a hardening at low observer inclinations and progressively softer spectra with increasing inclination, and will be somewhat independent of compact object (see for instance Karmakar et al. 2025). Given the association with super-Eddington accretion rates, it is unsurprising that similar unification models based on inclination-dependence have also been suggested for TDEs at early times (see Dai et al. 2018; Parkinson et al. 2025). Indeed, X-ray bright TDEs typically show thermal spectra which would be consistent with arising from an optically thick disc (with reprocessing of this emission down to lower energies) whilst at late times, changes in the spectrum may be associated with the outflows becoming optically thin (see Guolo et al. 2024, but see also Mummery et al. 2023).

It is important to note that, due to the large covering fraction of a thick disc with outflows, super-Eddington systems seen at high inclinations will *not* appear super-Eddington in terms of radiative luminosity; this affects all such systems including binaries (famously such as SS433: Begelman et al. 2006), AGN and TDEs (Dai et al. 2018, and possibly QPEs: Middleton et al. 2025). However, it remains possible to elucidate the super-Eddington nature of such systems should a measure for the kinetic luminosity be obtained (as has been the case for a number of concealed, assumed binary systems in nearby galaxies, e.g. Soria et al. 2014) which we expand upon below.

**Fig. 12:**
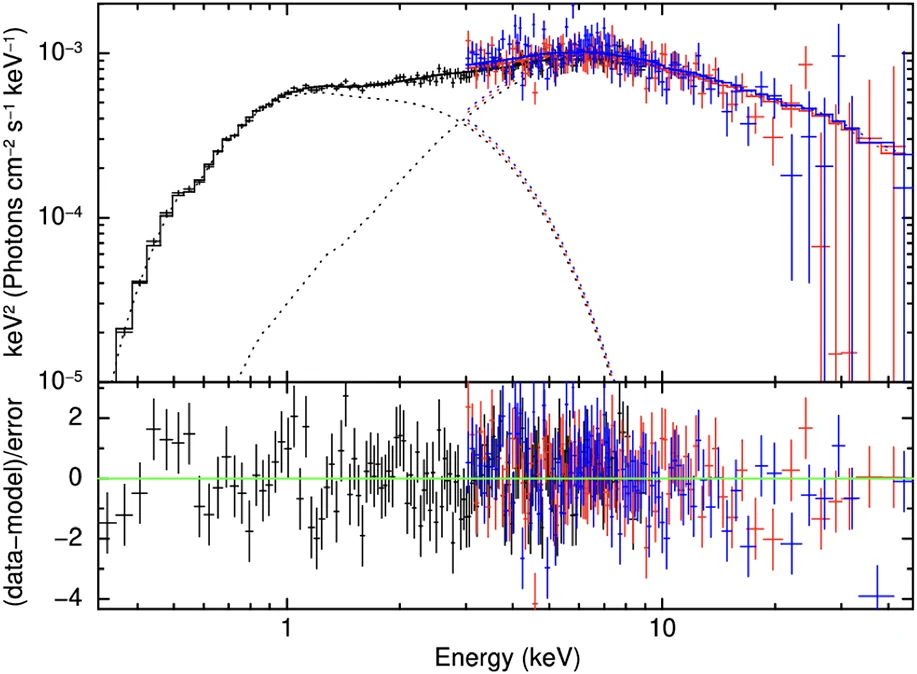
Plot from Mills et al. (2024) showing the joint XMM-Newton/NuSTAR data from ULX, NGC 1313 X-1 with a fitted model formed of an absorbed soft component (diskbb and a hard component which was extracted from a GRMHD simulation of super-Eddington accretion using Athena++. Residuals to the best fit are shown below

Finally, whilst there remain some issues extracting spectra from numerical simulations via post-processing (especially at high inclinations where line-of-sights can intercept regions which have not yet reached an equilibrium condition), there have been compelling attempts which some broad concordance with observation at low to moderate inclinations (Narayan et al. 2017; Dai et al. 2018; Mills et al. 2024 see Fig. 12).

### Outflows

A clear expectation from theory (those which are not entirely advection dominated) is that large scale height outflows should be present, as confirmed by all simulations to-date (see Sect. 2.3). These outflows have a polar angle dependence to both their velocities and densities. Naturally such outflows will be heated by the disc from multiple radii (and also from radiation advected with the flow) leading to a range of ionisation states. Outflows may be bound or unbound and potentially carry a large fraction of the liberated binding energy of the flow (equation (3)). Observationally, these outflows should leave their imprint spectrally and by imprinting variability due to scattering/obscuration (Middleton et al. 2011; 2015). Broad (CCD resolution) spectral features associated with outflows in ULXs were first identified by Middleton et al. (2014) and later confirmed (at higher energy resolution) by Pinto et al. (2016). Since that time, winds have been detected across all ULXs where data quality permits at soft energies (Pinto et al. 2017a, 2020, 2021; Kosec et al. 2021) and in once case at higher energies (Walton et al. 2016). Similar, fast winds have now been identified in both TDEs (Lin et al. 2015; Kara et al. 2018) and QPE sources (Kosec et al. 2025; Chakraborty et al. 2025), providing the tantalising opportunity to unify accretion across the mass scale (noting that the mass-scaled disc sizes in ULXs and TDEs/QPEs are likely to be very different due to the different feeding mechanisms).

**Fig. 13:**
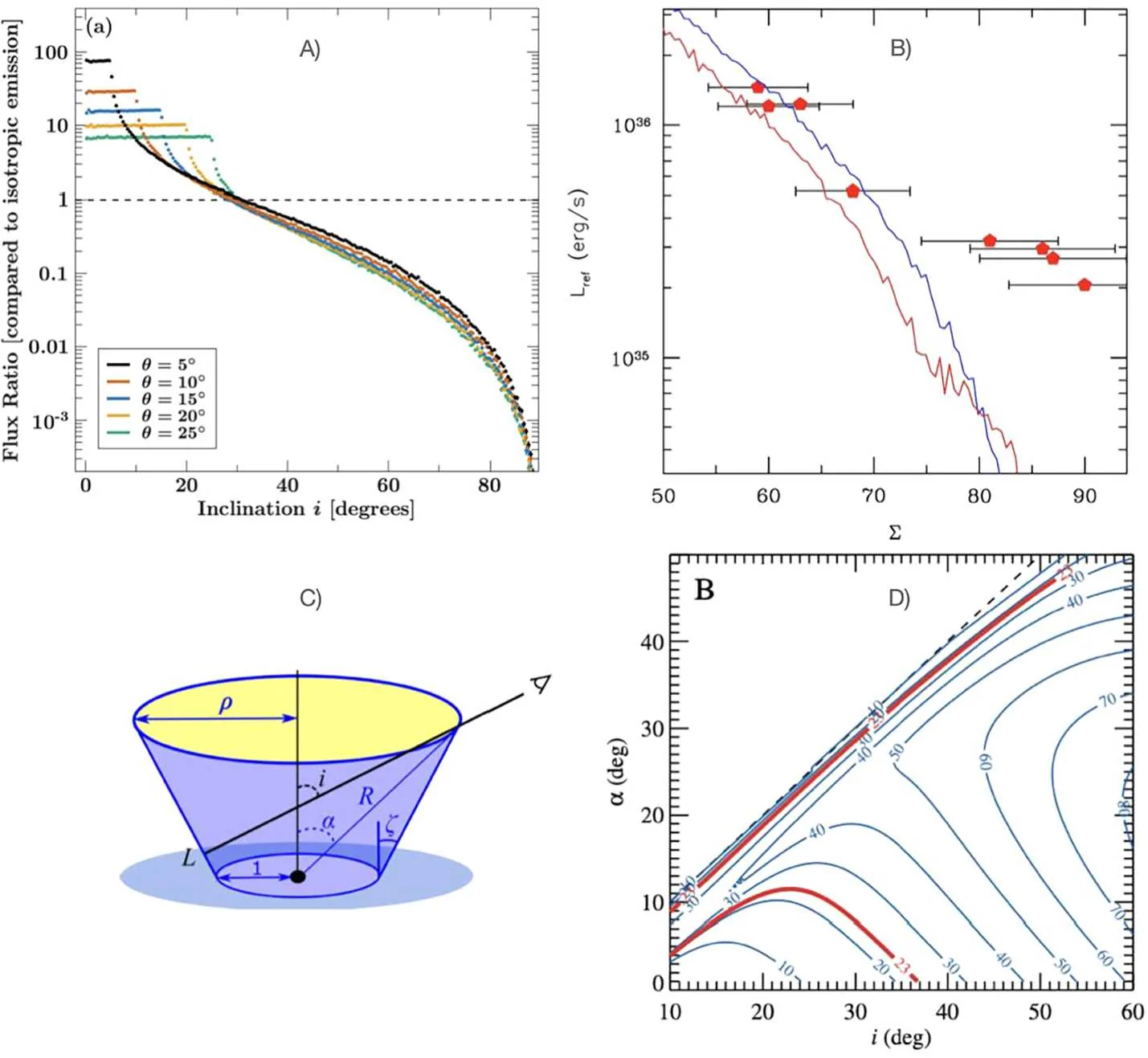
A) numerical simulation of the luminosity emerging from a conical geometry, as expected for super-Eddington accretion, by Dauser et al. (2017). The angle theta is the half-angle of the cone (α in C). It is apparent that there should be substantial collimation, leading to amplified, super-Eddington luminosities at small inclination and highly sub-Eddington luminosities outside of the cone. B) the model in A) was compared to the luminosity emerging from SS433 (Middleton et al. 2021, Σ refers to the inclination of the cone to our line-of-sight) with the finding that this source would have super-Eddington luminosities if viewed face-on (as had long been suspected given its highly super-Eddington nature: Begelman et al. 2006). C) the approximate super-Eddington geometry assumed for Cyg X-3 which was confirmed using spectro-polarimetric data by IXPE, D) (Veledina et al. 2024) where the red lines indicate the polarised degree detected, and the constraints this places on the opening angle and inclination of the source

Assuming that the winds detected to-date are launched from a super-critical disc, the inevitable conclusion is that there must be some level of collimation of radiation that emerges from within the outermost launching radius, and which will have a profound effect on how we both discover and interpret super-critically accreting sources. Numerical simulations imply that the amount of collimation depends on the accretion rate to some extent (Jiang et al. 2019), although locating the complete photosphere in simulations, especially at very high rates can be extremely challenging (although see e.g. Thomsen et al. 2022b) leading to a well-defined inclination dependence for the radiative luminosity (see following section for the impact of the emergent SED). Attempts to obtain the inclination dependence numerically via Monte Carlo simulations (Dauser et al. 2017 - see Fig. 13) have proven valuable and roughly match the predicted 1/(1-cos(θ)) dependence (Fukue 2011). Such simple simulations cannot capture the full complexity of the SED nor optical depth effects but can be instructive for first principles comparison to sources which are suspected to be highly super-critical and precess, changing our instantaneous line-of-sight inclination to the obscured innermost regions. As examples both SS433 and Cyg X-3 have been studied, the former using NuSTAR data to elucidate the phase dependent reflected emission (Middleton et al. 2021, Fig. 13), and the latter using X-ray polarisation data from IXPE (Veledina et al. 2024) which has provided an independent indication of the presence of the predicted geometry for a supercritical disc (Fig. 13).

### Nebulae

A further method for testing the role of collimation (or lack thereof) and constraining the kinetic luminosity of super-critical sources is to study the surrounding material on large scales. The most well-studied examples are the bubble nebulae which have been located around a limited number of ULXs and which are considerably larger than a natal SNR (Pakull and Mirioni 2002). Assuming the ULX nebula is inflated by outflows, their sizes (recognising that they are not truly spherical) allow the input kinetic energy to be estimated (see Weaver et al. 1977), with values obtained in the region of 10^39^ - 10^40^ erg/s (e.g. Cseh et al. 2012).

The response of photo-ionised recombination lines (predominantly HeII 4686λ) to an unseen SED can be used to infer the isotropic radiation field seen by the material, and was suggested as a method to constrain the amount of beamed emission from ULXs (Pakull and Mirioni 2002). However, as shown by Beuchert et al. (2024), it is important to stress that such nebulae are not as sensitive to hard X-ray emission as they are to the UV-soft X-ray emission. As a result the fact that the inferred illuminating radiative luminosity is similar to that observed from soft ULXs, does not confer a lack of beaming at harder energies.

### The Role of Advection

Numerical simulations (see Sect. 2.3) typically infer strong radial advection, with values for $L_{\mathrm{kin}}/L_{\mathrm{rad}}$ of less than unity (with some even below 0.01: Utsumi et al. 2022). Measurements of winds from ULXs and TDEs (see above) clearly enable estimates for $L_{\mathrm{kin}}$, requiring only the outflow velocity (noting that this is projected along the line-of-sight), ionisation state and covering fraction:

4 $$ L_{\mathrm{kin}} = 2\pi L_{\mathrm{ion}} \frac{\Omega}{\xi}v_{\mathrm{wind}}^{3}m_{ \mathrm{p}} $$

In the above, $v_{\mathrm{wind}}$ and ξ can be obtained by fitting models (collisionally and/or photoionised) with the latter responding to the assumed input SED ($L_{\mathrm{ion}}$), whilst the covering fraction (Ω) is somewhat unconstrained but is likely substantial given the ubiquity of signals now resolved into outflows (Kosec et al. 2021).

The anisotropy of the accretion flow demands that the softest emission be the least collimated (to which the nebula responds, see above) and should therefore provide the most reasonable estimate for the intrinsic $L_{\mathrm{rad}}$. Taking NGC 5408 X-1 as one of the best examples as it is both spectrally soft, has a nebula, and has well-modelled wind features, we find that $L_{\mathrm{kin}}/L_{\mathrm{rad}}$ to be >0.1 which becomes higher should the radiation be at all beamed (with other ULXs providing similar constraints or implying even higher ratios - see Table 1 of King, Lasota & Middleton 2023). Taken as a whole, this implies that inwards radial advection is likely to be a sub-dominant component of the energetics when the feeding radius is large. Such a conclusion would seem to match numerical simulations by Fragile et al. (2025) and modelling of the X-ray spectra of a transient ULX in M31 by Straub et al. (2013) where advection was not a requirement at the highest (near-Eddington) luminosities.

## Summary and Thoughts on How Progress Might Be Made

The overall structure and influence of super-Eddington accretion flows is becoming better understood as a result of observation. Such progress is well timed as we are entering a period where post-processing of numerical simulations is becoming feasible, permitting direct tests of our grasp of the underlying theory. However, there remain a number of outstanding issues which obscure a more complete understanding of super-Eddington accretion; addressing them cannot rely on isolating theory and observation but requires the synergy of the two.

One of the outstanding questions which plagues not only this area of accretion physics but more generally is whether the flow is MAD (magnetically dominated) or not. Numerical simulations imply there should be marked differences in the strength of the wind (which will be amplified relative to only radiation pressure driving) and the presence of a Blandford-Znajek type jet, as well as a likely impact on the global dynamics (see Fragile et al. 2023). There also appear marked differences in the results of numerical simulations depending on how material is fed (cf Jiang et al. 2014; Fragile et al. 2025) with implications for the role of advection; this then raises the question of whether there are cases in nature where certain conditions might prevail (e.g. TDEs versus ULXs) nd how much mass makes it to the BH. It would seem likely that testing this potentially conflicting picture requires clear observational predictions (time dependent, multi-wavelength, spectro-polarimetric) and a comparison to data. Fortunately with the advent of highly optimised codebases for GPU deployment (e.g. AthenaK: Stone et al. 2026), we can hope to run simulations for longer physical timescales in the disc and obtain families of post-processed data products (see also ‘Future Work’ in Sect. 2.3).

The future looks bright for studying super-Eddington accretion observationally. In the immediate future we can hope that XRISM may provide a deeper view of outflows (should the Resolve valve gate open) and that LSST will locate large numbers of TDEs as ZTF has done so. In the longer term, NewAthena will undoubtedly herald a far more complete picture of how the accretion flow operates in super-Eddington sources, from the inclination dependence of outflows to the discovery of many more pulsing neutron star systems. We must also remember that both ULXs and TDEs (and super-Eddington AGN such as some narrow line Seyfert 1s) emit across a broad swathe of energies and SKA will no doubt detect many such systems with bright jets (as well as termination shocks), helping us test the energy content in the jets (and thereby testing MAD scenarios). Taken as a whole, observations will help us constrain the energies of specific components (winds, jet and radiation) and test our theoretical understanding.

## Data Availability

Not applicable

## Footnotes

1 In the super-Eddington disc with both wind and advection (Lipunova 1999), the slope of the density profile ρ(r) varies between the ${-3/2}$ of the ADAF and ${-1/2}$ of the super-Eddington model of Shakura and Sunyaev (1973).
